# Association Between 5HT1b Receptor Gene and Methamphetamine Dependence

**DOI:** 10.2174/157015911795017137

**Published:** 2011-03

**Authors:** H Ujike, M Kishimoto, Y Okahisa, M Kodama, M Takaki, T Inada, N Uchimura, M Yamada, N Iwata, M Iyo, I Sora, N Ozaki

**Affiliations:** 1 Department of Neuropsychiatry, Okayama University Graduate School of Medicine, Dentistry and Pharmaceutical Sciences, Okayama, Japan; 2 JGIDA (Japanese Genetics Initiative for Drug Abuse), Japan; 3 Seiwa Hospital, Tokyo, Japan; 4 Department of Neuropsychiatry, Kurume University Graduate School of Medicine, Kurume, Japan; 5 Department of Psychogeriatrics, National Institute of Mental Health, National Center of Neurology and Psychiatry, Kodaira, Japan; 6 Department of Psychiatry, Fujita Health University School of Medicine, Houmei, Japan; 7 Department of Psychiatry, Chiba University Graduate School of Medicine, Chiba, Japan; 8 Department of Neuroscience, Division of Psychobiology, Tohoku University Graduate School of Medicine, Sendai, Japan; 9 Department of Psychiatry, Nagoya University Graduate School of Medicine, Nagoya, Japan

**Keywords:** Methamphetamine dependence, association study, *HTR1B*, haplotype.

## Abstract

Several lines of evidence implicate serotonergic dysfunction in diverse psychiatric disorders including anxiety, depression, and drug abuse. Mice with a knock-out of the 5HT1b receptor gene (*HTR1B*) displayed increased locomotor response to cocaine and elevated motivation to self-administer cocaine and alcohol. Previous genetic studies showed significant associations of *HTR1B* with alcohol dependence and substance abuse, but were followed by inconsistent results. We examined a case-control genetic association study of *HTR1B* with methamphetamine-dependence patients in a Japanese population. The subjects were 231 patients with methamphetamine dependence, 214 of whom had a co-morbidity of methamphetamine psychosis, and 248 age- and sex-matched healthy controls. The three single nucleotide polymorphisms (SNPs), rs130058 (A-165T), rs1228814 (A-700C) and rs1228814 (A+1180G) of *HTR1B* were genotyped. There was no significant difference in allelic and genotypic distributions of the SNPs between methamphetamine dependence and the control. Genetic associations of *HTR1B* were tested with several clinical phenotypes of methamphetamine dependence and/or psychosis, such as age at first abuse, duration of latency from the first abuse to onset of psychosis, prognosis of psychosis after therapy, and complication of spontaneous relapse of psychotic state. There was, however, no asscocation between any SNP and the clinical phenotypes. Haplotype analyses showed the three SNPs examined were within linkage disequilibrium, which implied that the three SNPs covered the whole *HTR1B*, and distribution of estimated haplotype frequency was not different between the groups. The present findings may indicate that *HTR1B* does not play a major role in individual susceptibility to methamphetamine dependence or development of methamphetamine-induced psychosis.

## INTRODUCTION

Family and twin studies have provided evidence that genetic factors can influence individual differences in vulnerability to substance abuse and dependence [1, 2]. We previously reported that patients with methamphetamine use disorders showed substantial individual differences in psychotomimetic and psychotogenic effects of methamphetamine consumption, e.g., intensity of subjective euphoric effects, latency to onset of methamphetamine-induced psychosis, and prognosis of psychosis after discontinuance of methamphetamine use [3], whose clinical variations should be affected by individual genetic background.

Pharmacological manipulation of serotonergic signaling can modulate the activity of brain reward pathways, and thus the effects of substance dependence to diverse classes of drugs. Fluoxetine, a selective serotonin uptake inhibitor, reduced self-administration of cocaine [4]. Ethanol intake decreased after the administration of 5-HT precursors, 5-HT uptake inhibitors, intracerebral 5-HT, and postsynaptic 5-HT agonists in animals and humans [5]. These serotonergic effects against drug abuse could be mediated by 5HT1b receptors at least. Administration of a 5HT1b agonist, CP-94,253, reduced ethanol self-administration and alcohol-induced aggressive behaviors *via* activation of postsynaptic 5HT1b receptors [6, 7]; in contrast, it facilitated cocaine reward by reducing 5HT release *via *5HT1b autoreceptor stimulation at presynaptic sites [8]. Mice lacking 5HT1b receptors displayed increased locomotor response to and self-administration of cocaine [9], and elevated alcohol consumption [10].

Previous genetic studies indicated that the 5HT1b receptor gene (*HTR1B*, MIM 182131) was associated with drug dependence and related behaviors. Thus, *HTR1B* polymorphisms were reported to be associated significantly with alcoholism with antisocial behaviors [11, 12], whole alcoholism [13, 14], substance dependence [15] and heroin addiction [16], although there were also several inconsistent reports [17, 18]. Therefore, in order to investigate the roles of *HTR1B* in substance dependence, we examined a possible genetic association of *HTR1B* with methamphetamine dependence in a Japanese population.

## METHODS

### Subjects

The subjects consisted of 231 patients with methamphetamine dependence (184 male, 47 female; mean age±SD, 36.6±11.8) and 248 age-, sex-, and geographical origin-matched healthy controls (198 male, 50 female; mean age±SD, 36.6±10.6), who have no individual or family history of drug dependence or major psychotic disorders such as schizophrenia and bipolar disorders. Almost all patients (N=214) are or were co-morbid with methamphetamine-induced psychosis. All subjects were unrelated Japanese. Consensus diagnoses of methamphetamine dependence were made by two trained psychiatrists according to the ICD-10 criteria on the basis of interviews and medical records. The study protocol and purpose were explained to all subjects participating in the study, and written informed consent was obtained from all subjects. This study was approved by the Ethics Committee of each participating institute of Japanese Genetics Initiative for Drug Abuse (JGIDA) [19].

The patients with methamphetamine dependence and/or psychosis were divided into subgroups according to several clinical phenotypes that may indicate indirectly the severity of and liability to dependence and psychosis: 1) age at first abuse of methamphetamine: younger than 20 years old, which is underage in Japan, or older; 2) latency to onset of psychotic state after initial methamphetamine consumption: divided into two groups by median latency of 3 years; 3) Duration of psychotic state after discontinuance of abuse and therapy with antipsychotics: transient type and prolonged type, which were defined as psychosis that subsides within one month or lasts longer than one month, respectively; 4) complication of spontaneous psychosis after remission of methamphetamine-induced psychosis, and 5) multi-substance abuse status.

### Genotyping

*HTR1B* consists of single exon and is a relatively small gene of about 11.7 Kb. HapMap data indicates that there is only one single nucleotide polymorphism (SNP) in the exon, rs 6297, which was proven polymorphic in a Japanese population. However, we did not examine this SNP because it is synonymous, Val3Val, indicating no or less physiological involvement. Instead, we genotyped three SNPs flanking the the gene, rs6297 (A-700C) and rs130058 (A-161T) in the 5’ flanking region and rs1228814 (A+1180G) in the 3’ flanking region, which have potential to be functional. Genotyping was performed by the PCR-RFLP method. The genomic DNA was extracted from peripheral leukocytes using a standard method. Each polymorphic site was amplified by PCR (the PCR primer sequence of each SNP is available on request) in a 15-ml volume containing 3% dimethyl sulfoxide and 0.75 units of Taq DNA polymerase (Promega Co., Japan) using a unique primer set. The PCR reaction was performed under the following conditions: 95°C for 5 min, then 35 cycles of 30 s of denaturing at 95°C, 1 min of annealing at the appropriate temperature, and 30 s of extension, and final elongation at 72°C for 10 min. The PCR products were digested with the corresponding restriction enzyme for each polymorphism, Nde*I* for rs6297, Nla*III* for rs130058 and Cfro13*I* for rs1228814, and then electrophoresed on 3.0% agarose gels and stained with GelStar (Takara Co., Japan). All genotyping was performed in a blinded fashion, with the control and cases samples mixed randomly. The genotyping of the SNPs were confirmed in part by direct sequencing.

### Statistical Analysis

Statistical analysis of association was performed using SNPAlyze software (Dynacom Co., Japan). Deviation from Hardy-Weinberg equilibrium and the case-control study were tested using the χ^2^ test for goodness of fit and χ^2^ test for dependence, respectively. Linkage disequilibrium (LD) was tested using the χ^ 2^ test, and D’ and r^2^ values were made the index in the authorization of LD. Case-control haplotype analysis was performed by the permutation method, and permutation *p*-values were calculated based on 100,000 replications.

## RESULTS

The genotype distribution and allele frequencies of the each polymorphism are shown in Table **1**. The genotype distributions of patients and control subjects did not deviate from Hardy-Weinberg equilibrium at any SNP examined. We found no significant difference between the patients and controls in the frequencies of the genotype or allele at any SNP of *HTR1B* (rs6297: allele: p=0.37, genotype: p=0.38, rs130058: allele: p=0.30, genotype: p=0.33 rs1228814: allele: p=0.14, genotype: p=0.47).

We estimated the pairwise LD between the three SNPs of *HTR1B* using the D’ and r^2 ^values as an index. A D’ of more than 0.7 was found between all the SNPs (0.8455 between rs6297 and rs130058, 1.000 between rs6297 and rs1228814, 0.8216 between rs130058 and rs1228814) indicating that the three SNPs are in linkage disequilibrium (LD) and located within one LD block. Then, we performed case-control haplotype analysis (Table **2**). There were 5 kinds of haplotypes consisting of the three SNPs. There was no significant difference in distribution of haplotype between methamphetamine dependence and controls (overall permutation *p*=0.81). Neither haplotype consisting of the two SNPs in the promoter region (rs6297 and rs130058) showed a significant difference in distribution between the groups.

Additional analyses of subgroups of patients with methamphetamine dependence/psychosis stratified by five items of clinical phenotypes (Table **3**) revealed that there was no significant association of any SNP of *HTR1B* with any clinical phenotype of methamphetamine dependence and/or psychosis.

## DISCUSSION

The 5HT1b receptors are expressed in the brain of rodents, and homologous 5HT1Dβ receptors are expressed in the human brain. The 5HT1b receptors are located at nerve terminals of various pathways and act as autoreceptors that are involved in the regulation of release of diverse neurotransmitters, including serotonin itself [20]. The 5HT1b receptors are also located at postsynaptic sites. A lot of studies suggest that 5HT1b receptors are implicated in several physiological functions, behaviors, and neuropsychiatric disorders including migraine, aggression, anxiety, depression, and substance dependence [20].

Genetic associations of HTR1B have been examined with various psychiatric conditions such as antisocial behaviors, suicide, depression, and schizophrenia. As to substance dependence, Lappapleinene *et al*. [11] found that rs6296, a synonymous SNP in exon 1 (G861C, Val3Val), was associated with antisocial alcoholism in two independent populations of alcoholic patients for the first time, but this was followed by consistent [12] and inconsistent results [15, 17, 18, 21]. Finally, Fehr *et al*. [13] reported that the risk allele of 861C reported by Lappapleinene *et al*. [11] was protective in their patients with alcoholism. These inconsistencies among alcoholism studies may indicate that status of comorbidity with other substance abuse could influence the results because *HTR1B* was shown to be associated with substance abuse [15] and heroin addiction [16].

In the present study, we examined three SNPs in the 5’ and 3’ flanking regions of *HTR1B*, rs6297 (A-700C), rs130058 (A-161T), and rs1228814 (A+1180G) in patients with methamphetamine dependence and found no association at any loci. Neither was any association found with several clinical phenotypes, such as initial abuse of methamphetamine at a younger age, rapid onset of psychotic state induced by methamphetamine, longer duration of psychosis after discontinuance of methamphetamine abuse, complication of spontaneous relapse of psychosis after remission, and multisubstance abuse status. Haplotype analysis of the three SNPs also showed no significant difference in haplotype distribution between the patients and controls. As the LD block consisting of the three SNPs covers the whole of *HTR1B*, it is unlikely that any untested polymorphism including G861C in *HTR1B* could be associated with methamphetamine dependence or its clinical phenotypes. Our findings are consisted with a study of cocaine, another psychostimulant, which showed that T-261G, A-161T, and G861C of *HTR1B* was not associated with cocaine abuse [17].

Duan *et al.* [22] examined effects of common SNPs in the promoter region of *HTR1B* on its transcription activity by *in vitro* reporter assay and revealed that T-261G and A-161T (rs130058) potently affected gene expression. The haplotypes consisting of -261G and -161A enhanced transcriptional activity 2.3-fold compared with major haplotype consisting of -261T and -161T. The A-161T polymorphism altered characteristics of binding to AP-1 transcription factor. Therefore, our negative findings may be significant and indicate that higher or lower density of the 5HT1b receptor due possession of -161A or -161T of *HTR1B* does not affect individual susceptibility to methamphetamine dependence and psychosis.

## Figures and Tables

**Table 1.** Case-Control Association Analyses of *HTR1B*

Loci Groups	N		Genotype (%)		*p*	Allele (%)		*p*
SNP1 (rs6297)		A/A	A/G	G/G		A	G	
Control	248	73.4	23.8	2.8		85.3	14.7	
MAP-dependence	228	68.9	27.6	3.5	0.37	82.7	17.3	0.38
SNP2 (rs130058)		T/T	T/A	A/A		T	A	
Control	227	87.2	12.8	0		93.6	6.4	
MAP-dependence	229	89.5	10.5	0	0.3	94.8	5.2	0.33
SNP3 (rs1228814)		C/C	C/A	A/A		C	A	
Control	246	73.6	25.2	1.2		86.2	13.8	
MAP-dependence	225	70.7	27.5	1.8	0.14	84.4	15.6	0.47

**Table 2.** Haplotype Analysis of *HTR1B* in Methamphetamine Dependence

Haplotype	Controls	MAP-Dependence	*p*
A-T-C	0.6973	0.6721	0.41
G-T-C	0.1480	0.1673	0.43
A-T-A	0.0902	0.1095	0.33
A-A-A	0.0520	0.0461	0.68
A-A-C	0.0094	0.0034	0.26

Global permutation *p* value =0.81 (c^2^=3.20).

**Table 3.** Association of *HTR1B* with Clinical Phenotypes of Methamphetamine Dependence and Psychosis

SNP1 (rs6297)	N		Genotype (%)		*p*	Allele (%)		*p*
		A/A	A/G	G/G		A	G	
Age at first use								
20y <=	111	0.68	0.26	0.05		0.82	0.18	
19y >=	113	0.68	0.30	0.02	0.37	0.83	0.17	0.38
Latency of psychosis								
3y >	103	0.64	0.31	0.05		0.80	0.20	
3y <=	83	0.70	0.28	0.02	0.57	0.84	0.16	0.31
Prognosis of psychosis								
Transient	114	0.68	0.30	0.03		0.82	0.18	
Prolonged	84	0.69	0.26	0.05	0.65	0.82	0.36	0.94
Spontaneous relapse of psychotic symptoms								
+	84	0.62	0.36	0.02		0.80	0.20	
－	129	0.71	0.24	0.05	0.15	0.83	0.17	0.35
Poly-substance abuse								
+	158	0.69	0.28	0.03		0.83	0.17	
－	63	0.68	0.27	0.05	0.85	0.82	0.18	0.77
**SNP2 (rs130058)**	**N**	**Genotype (%)**			***p***	**Allele (%)**		***p***
		**T/T**	**T/A**	**A/A**		**T**	**A**	
Age at first use								
20y <=	111	0.86	0.14	0.00		0.93	0.07	
19y >=	114	0.92	0.08	0.00	0.31	0.96	0.04	0.33
Latency of psychosis								
3y >	103	0.91	0.09	0.00		0.96	0.04	
3y <=	84	0.90	0.10	0.00	0.85	0.95	0.05	0.86
Prognosis of psychosis								
Transient	115	0.88	0.12	0.00		0.94	0.06	
Prolonged	84	0.93	0.07	0.00	0.24	0.96	0.04	0.26
Spontaneous relapse of psychotic symptoms								
+	85	0.92	0.08	0.00		0.96	0.04	
－	129	0.87	0.13	0.00	0.26	0.93	0.07	0.28
Poly-substance abuse								
+	159	0.90	0.10	0.00		0.95	0.05	
－	63	0.87	0.13	0.00	0.57	0.94	0.06	0.58
**SNP3 (rs1228814)**	**N**	**Genotype (%)**			***p***	**Allele (%) **		***p***
		**C/C**	**C/A**	**A/A**		**C**	**A**	
Age at first use								
20y <=	110	0.72	0.25	0.04		0.84	0.16	
19y >=	111	0.68	0.32	0.00	0.14	0.84	0.16	0.47
Latency of psychosis								
3y >	100	0.76	0.22	0.02		0.87	0.13	
3y <=	83	0.66	0.33	0.01	0.27	0.83	0.17	0.23
Prognosis of psychosis								
Transient	113	0.71	0.28	0.01		0.85	0.15	
Prolonged	82	0.73	0.24	0.02	0.59	0.85	0.15	0.91
Spontaneous relapse of psychotic symptoms								
+	81	0.73	0.27	0.00		0.86	0.14	
－	129	0.69	0.28	0.03	0.27	0.83	0.17	0.34
Poly-substance abuse								
+	156	0.70	0.28	0.02		0.84	0.16	
－	62	0.69	0.29	0.02	0.98	0.84	0.16	0.98
